# Enhanced rhamnolipid production in *Burkholderia thailandensis* transposon knockout strains deficient in polyhydroxyalkanoate (PHA) synthesis

**DOI:** 10.1007/s00253-017-8540-x

**Published:** 2017-10-17

**Authors:** Scott J. Funston, Konstantina Tsaousi, Thomas J. Smyth, Matthew S. Twigg, Roger Marchant, Ibrahim M. Banat

**Affiliations:** 1 School of Biomedical Sciences, Ulster University, BT521SA, Coleraine, Northern Ireland UK; 2 Department of Life Sciences, Institute of Technology Sligo, Sligo, County Sligo Ireland

**Keywords:** Rhamnolipid, *Burkholderia thailandensis*, PHA, Knockout strains

## Abstract

Microbially produced rhamnolipids have significant commercial potential; however, the main bacterial producer, *Pseudomonas aeruginosa*, is an opportunistic human pathogen, which limits biotechnological exploitation. The non-pathogenic species *Burkholderia thailandensis* produces rhamnolipids; however, yield is relatively low. The aim of this study was to determine whether rhamnolipid production could be increased in *Burkholderia thailandensis* through mutation of genes responsible for the synthesis of the storage material polyhydroxyalkanoate (PHA), thereby increasing cellular resources for the production of rhamnolipids. Potential PHA target genes were identified in *B. thailandensis* through comparison with known function genes in *Pseudomonas aeruginosa*. Multiple knockout strains for the *phbA*, *phbB* and *phbC* genes were obtained and their growth characteristics and rhamnolipid and PHA production determined. The wild-type strain and an rhamnolipid (RL)-deficient strain were used as controls. Three knockout strains (Δ*phbA*1, Δ*phbB*1 and Δ*phbC*1) with the best enhancement of rhamnolipid production were selected for detailed study. Δ*phbB*1 produced the highest level of purified RL (3.78 g l^−1^) compared to the wild-type strain (1.28 g l^−1^). In Δ*phbB*1, the proportion of mono-rhamnolipid was also increased compared to the wild-type strain. The production of PHA was reduced by at least 80% in all three *phb* mutant strains, although never completely eliminated. These results suggest that, in contrast to *Pseudomonas aeruginosa*, knockout of the PHA synthesis pathway in *Burkholderia thailandensis* could be used to increase rhamnolipid production. The evidence of residual PHA production in the *phb* mutant strains suggests *B. thailandensis* possesses a secondary unelucidated PHA synthesis pathway.

**Electronic supplementary material:**

The online version of this article (10.1007/s00253-017-8540-x) contains supplementary material, which is available to authorized users.

## Introduction

There is considerable current industrial interest in microbial biosurfactants as replacements for chemical surfactants in a wide range of commercial products including food (Campos et al. 2013), pharmaceutical (Fracchia et al. 2015), health (Elshikh et al. 2016), petroleum (De Almeida et al. 2016) and as general replacement to chemical surfactants (Marchant and Banat 2012b). The route to exploitation has so far not been without difficulties; however, there are now a number of companies that are bringing microbial-produced biosurfactant to the market (Marchant and Banat 2012a). The main issues have been focussed around obtaining sufficient yield from microbial fermentation coupled with the need to find cost-effective downstream processing strategies to produce a commercially viable final product competitive with the chemical surfactants in current use. Although there is a wide range of different biosurfactants produced by bacteria, fungi and yeasts, the low molecular weight glycolipid biosurfactants have attracted the most attention and within this group the rhamnolipids have been investigated extensively. The first identified and most widely studied rhamnolipid producer is *Pseudomonas aeruginosa*; however, this bacterium is a known opportunistic pathogen which precludes industrial exploitation as a biosurfactant-producing organism. A potential solution to this problem has been to seek alternative naturally occurring non-pathogenic microorganisms that could be used in place of *P. aeruginosa* (Marchant et al. 2014)*.* One such organism is *Burkholderia thailandensis* which produces predominantly di-rhamnolipid with C_14_C_14_ alkyl chains (Dubeau et al. 2009; Funston et al. 2016).

A review published by Müller and Hausmann (2011) proposed a systems biology approach to genetically ‘enhance’ the bacterial metabolome to maximise rhamnolipid production to an industrially viable scale using methods such as genetic alteration of existing rhamnolipid (RL) producers and recombinant production using heterologous hosts (Müller and Hausmann 2011). Several studies have been carried out on *P. aeruginosa* and other potential RL-producing strains in an attempt to produce a strain that is highly efficient in RL production. A study by Wang et al. (2007) showed that cloning the RL synthesis genes *rhlA* and *rhlB* into the non-RL-producing strains *P. aeruginosa* PAO1 Δ*rhlA* and *Escherichia coli* BL21(DE3) using transposon-mediated chromosome integration resulted in both newly engineered strains producing RL. The engineered *E. coli* BL21(DE3) strain was shown to produce only mono-RL demonstrating that genetic engineering can also be used to produce specific RL structures reducing downstream processing and purification stages (Wang et al. 2007). Zhao et al. (2015) showed the successful production of RL from *Pseudomonas stutzeri* DQ1 under anaerobic conditions after insertion of the *rhlABRI* operon. These studies show that heterologous production of RL is possible; however, RL production rates by these mutants (1.61 g l^−1^) are still too low to be regarded as industrially viable where an approximate yield of 4 g l^−1^ is required (Zhao et al. 2015). Finally, Grosso-Becerra et al. (2016) have attempted to address both the problem of the pathogenicity of *P. aeruginosa* and the yield of rhamnolipids by cloning some of the RL synthesis genes *rhlA* and *rhlB* into an apparently non-pathogenic strain *P. aeruginosa* ATCC 9027. This study showed that the recombinant strain was able to produce mono-rhamnolipid with a yield comparable to *P. aeruginosa* PAO1 and that the recombinant ATCC 9027 strain was non-pathogenic in a murine model (Grosso-Becerra et al. 2016).

A different approach to increase the RL yield is to genetically modify the metabolic processes within the bacterial cell to streamline RL production and therefore to artificially drive more resources towards RL production at the expense of other non-essential secondary metabolites. One such method of achieving this would be to eliminate the biosynthetic pathways of other secondary metabolites that are in direct competition for resources or precursors used in RL production. An example pathway is that of polyhydroxyalkanoic acid (PHA) synthesis. PHAs are linear polyesters produced by bacteria as intracellular carbon storage granules when carbon is present in excess. PHAs produced by *P. aeruginosa* primarily comprise medium-chain-length (MCL) monomers of between 6 and 14 carbon atoms. This diverse range in PHA monomer structure is thought to be dependent on the carbon source, specific PHA synthase enzymes and the metabolic pathways involved (Madison and Huisman 1999; Lee et al. 2004). A number of studies have demonstrated that the biosynthetic pathways of RLs and PHAs in *P. aeruginosa* are highly similar and compete for the same lipid precursors (Pham et al. 2004; Zhu and Rock 2008; Abdel-Mawgoud et al. 2014). In *P. aeruginosa*, the initial stage of RL synthesis is the conversion of R-3-hydrooxydecanoyl-CoA to β-D-(β-D-hydroxyalkanoyloxy)alkanoic acids (HHA) by RhlA (Zhu and Rock 2008). R-3-Hydrooxydecanoyl-CoA is primarily derived from the β-oxidation pathway of fatty acid synthesis through the actions of RhlYZ (Abdel-Mawgoud et al. 2014). It is this 3-hydroxyalkanoate pool that has been shown to be the precursor for PHA synthesis (Wang et al. 2012; Abdel-Mawgoud et al. 2014).

Lipid precursors for RL synthesis can also be derived from the de novo fatty acid biosynthesis pathway; here, the enzymes RhlA and PhaG compete for these precursors for the synthesis of RL and PHAs respectively (Zhu and Rock 2008). Studies by Rehm et al. (1998) and Fiedler et al. (2000) showed that in *P. aeruginosa* the *phaG* gene coding for (R)-3-hydroxyacyl-ACP:CoA transacylase plays an important role in linking the PHA and fatty acid synthesis pathways (Rehm et al. 1998; Fiedler et al. 2000). The *rhlA* gene coding for rhamnosyltransferase I in *P. aeruginosa* was also shown to directly compete with *phaG* for (R)-β-hydroxyalkanoyl-ACP, and in addition, *rhlA* was also shown to be able to produce CoA-linked fatty acid dimers using ACP-linked fatty acids (Soberón-Chávez et al. 2005; Cabrera-Valladares et al. 2006). This means that *rhlA* may also play a role in PHA synthesis in *P. aeruginosa*. This appears to be evident as *P. aeruginosa phaG* mutants are still capable of PHA production at a low level whereas in other RL-producing *Pseudomonas* spp. *phaG* mutants are completely deficient in PHA production (Gutierrez et al. 2013). This close relationship is evident in the structure of the RhlA and PhaG enzymes as there is a 57% DNA sequence homology between the two corresponding genes in *P. aeruginosa* PA14. In addition, both enzyme functions can be silenced using the same inhibitor compound, 2-bromohexanoic acid (2-BrHA), resulting in the inhibition of both RL and PHA simultaneously (Gutierrez et al. 2013).

It is clear from recent research that in *P. aeruginosa* the synthesis of PHA and RLs is closely linked with both synthetic processes competing for the same precursors. Although this presents a promising point for metabolic manipulation towards RL overproduction in *P. aeruginosa*, recent work has shown that this may prove difficult. A study by Choi et al. (2011) reported a range of mutant strains each with different PHA or RL synthesis genes knocked out. Results from this study showed that whilst the knockout of RL synthesis genes led to a significant increase in PHA production, this was not the case for RL production in strains where PHA synthesis genes had been knocked out (Choi et al. 2011). This result was further confirmed by the work of Abdel-Mawgoud et al. 2014; however, this study showed an increase in the ratio of mono-RL to di-RL upon inactivation of PHA synthesis (Abdel-Mawgoud et al. 2014). The lack of increased RL production in PHA synthesis mutants may be due to the limitation of rhamnose availability or connected with the stringent transcriptional control of RL synthesis that exists in *P. aeruginosa* mediated through various quorum-sensing and environmental regulatory systems which have been widely elucidated in previous research (Perfumo et al. 2013; Abdel-Mawgoud et al. 2014). In contrast to this, however, Torrego-Solana et al. (2013) showed that when the *phaC*1, *phaZ* and *phaC*2 genes were all knocked out together using site-directed mutagenesis in the same mutant, *P. aeruginosa* 47T2 ΔAD, a 28% increase in RL production was observed when grown using waste frying oils (2%) as a carbon source. This mutant also showed a higher rate of carbon consumption and an increase in the conversion efficiency of oleic acid to (E)-10-hydroxy-8-octadecanic acid highlighting the rearrangement of metabolic processes resulting from the PHA knockout (Torrego-Solana et al. 2012).

The species *Burkholderia thailandensis* is a known RL producer; however, information on PHA synthesis and its effect on RL production in this species is lacking (Funston et al. 2016). In this paper, we report on the PHA synthesis by *B. thailandensis* and demonstrate the effects on rhamnolipid production through the knockout of the *B. thailandensis* PHA synthesis genes.

## Materials and methods

### Microorganism and culture conditions

The wild-type organism used for this study was *Burkholderia thailandensis* E264 obtained from the American Type Culture Collection (ATCC 700388). The *B. thailandensis* E264 transposon mutants (Supplementary Table S1) were obtained from the University of Washington, USA (Gallagher et al. 2013). The rhamnolipid-deficient *B. thailandensis rhlA1/A2* double mutant was obtained from the University of Calgary Health Sciences Centre, Canada (Dubeau et al. 2009). All *Burkholderia* strains were maintained on either NB agar (*Oxoid*) or NB broth (*Oxoid*). Rhamnolipid and PHA production studies were carried out on a small scale. Seed cultures of *B. thailandensis* E264 were grown in 1-l Erlenmeyer flasks with 100 ml NB + 4% glycerol (*v*/*v*) at 30 °C with 200 rpm rotary shaking for 24 h. The OD_600 nm_ of the seed culture was adjusted to ~2.0 with sterile NB + 4% glycerol (*v*/*v*) before inoculation of batch fermentation flasks. Ten millilitres of this seed culture was then added to 90 ml sterile NB + 4% glycerol in a 1-L Erlenmeyer flask, and cultures were incubated at 30 °C with 200 rpm rotary shaking (Dubeau et al. 2009).

### Polymerase chain reaction

Polymerase chain reactions (PCR) were carried out in 0.2-ml PCR tubes and contained a final concentration of 1× PCR buffer (Invitrogen), 1.5 mM MgCl_2_, 0.2 mM of each d.NTP (Invitrogen), 0.5 μM forward and reverse primers, 50 ng template DNA and 1 U recombinant *Taq* DNA polymerase (Invitrogen). All PCR amplification was performed using a TC5000 Thermo cycler (Bibby Scientific Ltd., UK) and run under the following conditions: 1× initial denaturation cycle of 5 min at 95 °C, 30× cycles of denaturation at 95 °C for 30 s, annealing at 55–65 °C for 30 s and extension at 72 °C for 90 s followed by a final extension cycle at 72 °C for 10 min. Following amplification, PCR products were routinely held at 4 °C.

### Extraction, purification and quantification of rhamnolipid

Extraction of rhamnolipids from cell-free supernatant was carried out using acid precipitation followed by solvent extraction described by Smyth et al. (2010). Samples taken from fermentations were first centrifuged at 10,500×*g* for 15 min to remove the cells. Hydrochloric acid (Sigma-Aldrich) was used to adjust the pH of the cell-free supernatant to ~2.0. The supernatant was then extracted three times with an equal volume of HPLC-grade ethyl acetate (Sigma-Aldrich), and the aqueous phase was discarded. Anhydrous MgSO_4_ (Sigma-Aldrich) was then added at a concentration of 0.01 g ml^−1^ and mixed through the ethyl acetate to remove any residual aqueous phase. Ethyl acetate recovered from the extractions was filtered using grade 1 filter paper (Whatman) then dried under vacuum using a rotary evaporator (Buchi, Switzerland) leaving a crude honey-like RL extract. The sample was then dried again using nitrogen gas and weighed to give the RL crude extract yield.

Solid-phase extraction (SPE) was used to remove any impurities from the RL crude extract. Strata SI-1 Silica (55 μm, 70 Å) Giga tubes (Phenomenex) were used to separate and clean up the rhamnolipid containing crude extracts. HPLC-grade chloroform (Sigma-Aldrich) was passed through the SPE column until the silica was fully conditioned; the sample was then dissolved in a small amount of chloroform and applied to the column. Chloroform was then passed through the column until any contaminants had been washed out. A solvent solution of chloroform/methanol at a ratio of 5:0.3 was then used to elute the mono-rhamnolipids. A solvent solution of chloroform/methanol (ratio 5:0.5) was used to elute the di-rhamnolipids.

Purified samples for analysis by ESI-MS were first diluted to a concentration of 0.1 mg ml^−1^ in HPLC-grade methanol (Sigma-Aldrich). Direct infusions were carried out on a Thermo Spectra LCQ™ mass spectrometer fitted with a quadrupole ion trap. Rhamnolipid samples were analysed in ion negative mode with an acquisition range varying between 50 and 1000 Da. A spray voltage of 3.5 kV was used with a capillary temperature of 250 °C.

HPLC-QToF-MS of rhamnolipid samples was carried out after SPE purification. For HPLC separation, the following parameters were used: static phase, Agilent poroshell SB-C3, 2.1 × 100 mm, particle size 2.7 μm. Mobile phase 1, H_2_O (4 mM ammonium acetate), and mobile phase 2, MeCN, were used for chromatographic separation as follows: 0–17 min 50–70% mobile phase 2, 17.0–17.5 min 70% mobile phase 2, 17.5–18.0 min 70–50% mobile phase 2 and 18–20 min 50% mobile phase 2.

### Screening of bacterial strains for PHA production

*B. thailandensis* strains were initially screened for PHA production by staining colonies grown on NB agar (Oxoid) supplemented with 4% glycerol with a 0.02% (*w*/*v*) solution of Sudan Black in ethanol for 30 min at room temperature. Following staining, colonies were washed with 100% ethanol. Strains shown to be producing PHAs appeared bluish black in colour. Cultures of *P. aeruginosa* PAO1 and *E. coli* JM109 were used respectively as positive and negative controls in this assay (Liu et al. 1998).

### PHA extraction and quantification using GC-MS

PHA was extracted from *B. thailandensis* E264 cells using a method described by Guo et al. (2011) with some adjustments. Cells were collected by centrifugation of 50 ml liquid culture at 13,000×*g* for 15 min. The cell supernatant was discarded and the pellet lyophilised for 48 h. The cell dry weight was recorded and the lyophilised material extracted with chloroform and sodium hypochlorite (30 ml chloroform g^−1^ dry cell biomass with 3 ml g^−1^ 20% sodium hypochlorite solution *v*/*v* in d.H_2_O). Extraction took place over 24 h before centrifugation at 3000×g for 10 min. The chloroform phase was carefully removed and filtered using grade 1 filter paper (Whatman). The PHA was then precipitated using ice-cold methanol, and samples were dried completely using compressed nitrogen gas. The PHA extracts were weighed to obtain a crude extract yield before preparation for GC-MS analysis.

Methanolysis was carried out to prepare samples for GC-MS analysis as described by Wang et al. (2009). Chloroform was added at 400 μl mg^−1^ dry weight to dissolve the PHA, an equal volume of sulphuric acid:methanol solution (1.7:0.3) was added to it and the solution was incubated at 100 °C for 140 min. For the recovery and clean-up of methyl esters of PHA monomers, samples were cooled to room temperature and 2 ml 25% ammonia (Sigma-Aldrich) was added in a dropwise fashion until the sample could be safely vortexed for 2 min. Samples were then centrifuged at 3000×*g* for 5 min, and 1.5 ml of the chloroform phase was carefully collected and 500 μl d.H_2_O was added; the sample was then vortexed vigorously and centrifuged at 3000×*g* for 5 min. The chloroform phase was used for analysis.

GC-MS analysis was performed on an Agilent triple quadrupole equipped with an MS detector and fitted with an Agilent HP-5 ms column (30 m length, 0.25 mm internal diameter, 0.25 mm film) (Agilent Technologies, USA). The monomeric constituents of PHA polymers in the form of hydroxyalkanoic acid methyl esters and PHB as hydroxybutyric acid methyl esters were analysed. Methanolyzed samples (2 μl) were automatically injected into the GC at a split ratio of 1:50. Hexadecanoic acid was used as internal standard and was added before the methanolysis. The injection temperature was set at 280 °C while the oven and column temperatures were programmed as 60 °C for 1 min then increased to 120 °C at 20 °C min^−1^, and then increased to 250 °C at 15 °C min^−1^ and held for 5 min. Compressed helium was used as carrier gas. Mass spectra were acquired at 1250 scan speed using electron impact energy of 70 eV at 200 °C ion-source and 280 °C interface temperatures respectively. The PHAs were identified using the NIST database.

### Quantification of glycerol consumption during fermentations using GC-MS

Samples were taken for glycerol analysis by aseptically removing 1000 μl culture from the fermentation vessel. Cells were removed by centrifugation for 2 min at 10,500×*g*, and the cell supernatant was carefully transferred to a clean, sterile 1.5 ml Eppendorf tube and stored at − 20 °C for further analysis. Prior to GC-MS analysis, glycerol was first derivatised to glycerol triacetate and separated from the culture supernatant (Wu, et al. 2011). This was done by adding 10 μl NMIM (N-methylmidazole) to 10 μl culture supernatant in a 1.5-ml Eppendorf tube. A volume of 75 μl acetic anhydride was then added, and samples were incubated at room temperature for 5 min. After incubation, 100 μl dH2O was added and the sample was vortexed for 10 s. Dichloromethane was then added at a volume of 100 μl, and 10 μl hexadecane was also added as an internal standard. The sample was vortexed briefly and left to separate at room temperature. The organic phase was then collected, and 0.1 g Na_2_SO4 (anhydrous) was added to remove any residual aqueous phase from the sample. Samples were then filtered using grade 1 filter paper (Whatman® qualitative filter paper, grade 1) and added to clean, labelled HPLC vials (Sigma-Aldrich).

GC analysis was performed on an Agilent system equipped with an MS detector (Agilent Technologies, CA, USA). An HP-5 silica-based capillary column (30 m × 0.25 mm × 0.25 μm) was used for the separation of the glycerol derivative with a split ratio of 50:1. Compressed helium was used as the carrier gas at a flow rate of 1 ml min^−1^ in constant-flow mode. The initial column temperature was 140 °C for 2 min which then increased to 250 °C at a rate of 20 °C min^−1^ and maintained for 2 min. The inlet temperature was set at 210 °C and the detector temperature was 250 °C.

## Results

### Quantitative analysis of PHAs produced by *B. thailandensis* E264 using GC-MS

**Fig. 1:**
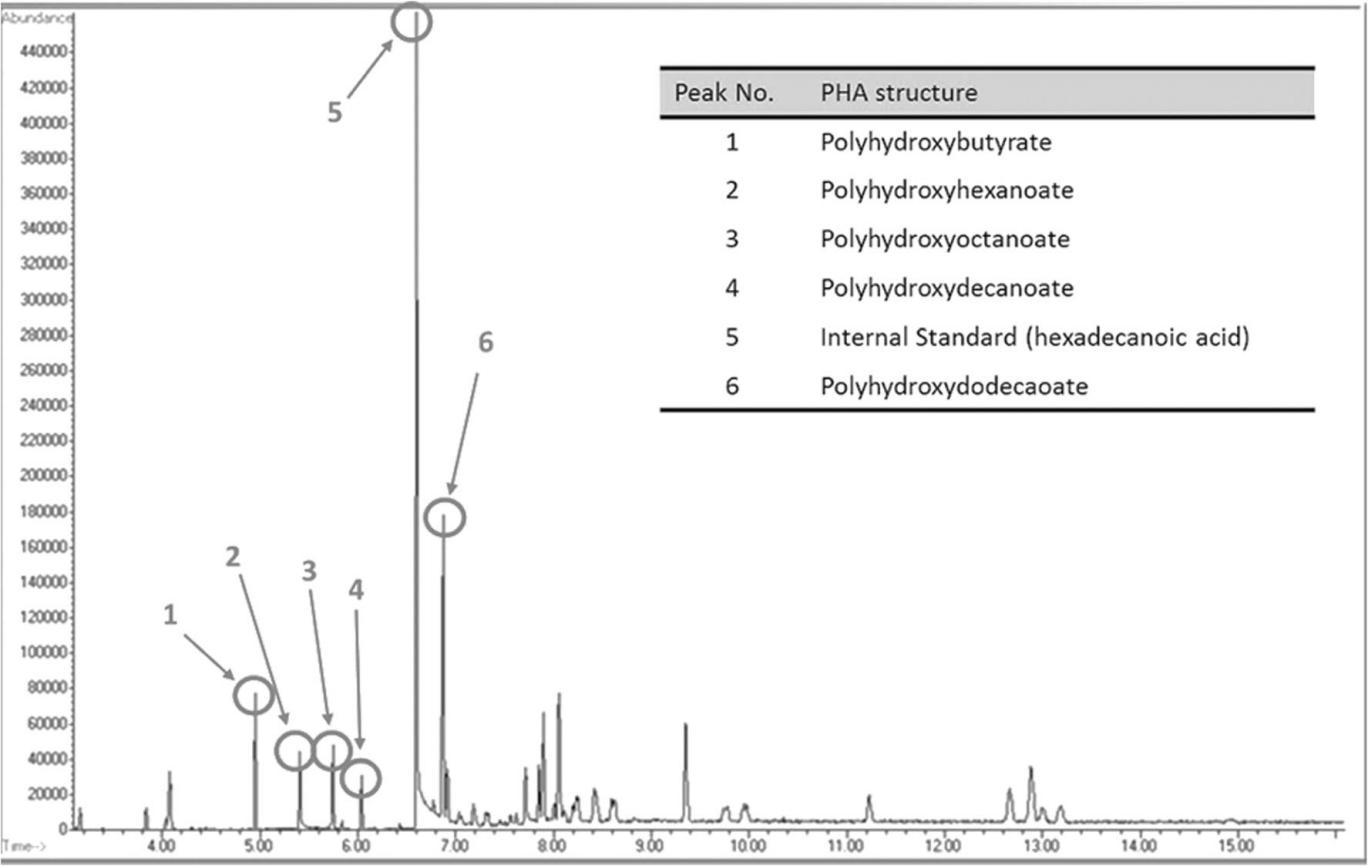
GC-MS chromatogram showing specific PHA monomers produced by WT *B. thailandensis* E264 (peaks 1–4, 6) and a hexadecanoic acid internal standard (peak 5) used for direct quantification of each PHA monomer

There have been no previous reports of PHA production within *B. thailandensis*; therefore, *B. thailandensis* E264 was screened for PHA production using Sudan Black staining. Following staining, *B. thailandensis* E264 appeared bluish black in colour suggesting PHA production (Supplementary Fig. S1). PHA production was further investigated by chemical analysis of *B. thailandensis* cultures. Any PHAs produced by *B. thailandensis* E264 shake flask cultures were extracted and analysed by GC-MS using protocols previously developed for PHA analysis in *P. aeruginosa*. GC-MS analysis of *B. thailandensis* E264 culture extracts showed the presence of hydroxyalkanoic acids (the monomeric units that form PHAs) corresponding to polyhydroxybutyrate, polyhydroxyhexanoate (C6), polyhydroxyoctanoate (C8), polyhydroxydecanoate (C10) and polyhydroxydodecanoate (C12) (Fig. 1).

### Identification of genes associated with PHA production in *B. thailandensis* E264

Due to the novel identification of the monomeric units that form PHAs in *B. thailandensis* cultures, the identification of putative PHA synthesis genes was considered paramount in establishing a potential mechanism for PHA synthesis in this species. There have been reports of PHA production in *Burkholderia sacchari* and other uncharacterised strains such as *Burkholderia* sp. USM (JCM15050) (Chee et al. 2010; Mendonça et al. 2014). Unfortunately, the genomic information and gene annotations for these organisms are limited and therefore unreliable to use for identification of PHA synthesis genes in *B. thailandensis*. *P. aeruginosa* is a known producer of PHA, and fully annotated genomic and proteomic sequence data for various strains are readily available (Hoffmann 2004; Pham et al. 2004). As such, it was decided to use *P. aeruginosa* as a model organism for PHA analysis in *B. thailandensis*.

**Fig. 2:**
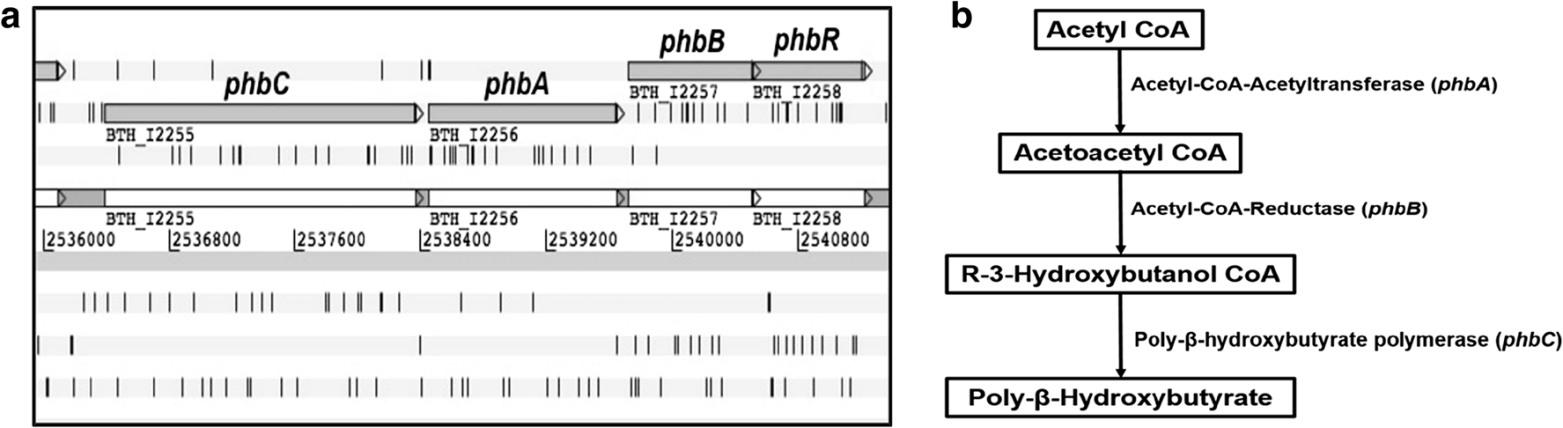
**a** Putative PHA synthesis gene cluster within the genome *B. thailandensis* E264, identified using the *Burkholderia* genome database (Winsor et al. 2011). BTH_I2255 (*phbC*) encodes a poly-β-hydroxybutyrate polymerase, BTH_I2256 (*phbA*) encodes an acetyl CoA-acetyltransferase, BTH_I2257 (*phbB*) encodes an acetyl CoA-reductase and BTH_I2258 (*phbR*) encodes a hypothetical protein predicted to be involved with the regulation of PHA synthesis. **b** The proposed biochemical pathway for the synthesis of PHA in *B. thailandensis*

In *P. aeruginosa*, the primary genes associated with PHA synthesis are *phaC1* and *phaC2* which code for poly(3-hydroxyalkanoic acid) synthase 1 and poly(3-hydroxyalkanoic acid) synthase 2 respectively **(**Hoffmann et al. 2000). In addition, *phaG* which codes for the enzyme (R)-3-hydroxydecanoyl-ACP:CoA transacylase was also found to play a major role in PHA synthesis (Hoffmann et al. 2000). The peptide sequences expressed from *phaC1*, *phaC2* and *phaG* in *P. aeruginosa* PAO1 were obtained from the NCBI. To determine putative PHA synthesis within *B. thailandensis*, a BLASTp analysis was carried out using these sequences against the *B. thailandensis* E264 genome (taxid:271848). Results from the BLAST search showed that PhaC1 and PhaC2 possessed sequence similarity with a poly-beta-hydroxyalkanoate polymerase found in *B. thailandensis* (Accession No: WP_019254714.1) with a percentage identity of 40 and 39% respectively and an 86 and 39% query coverage. The two identical RhlA homologues present in *B. thailandensis* proved to have the highest sequence identity with PhaG (sequence identity of 43% and a 91% query coverage). Further in silico analysis showed that the poly-beta-hydroxyalkanoate polymerase within *B. thailandensis* to which both PhaC1 and PhaC2 showed similarity is encoded by a gene annotated as *phbC* and that within the *B. thailandensis* genome this gene is given the locus tag BTH_I2255. Following closer examination of *phbC* within the *B. thailandensis* E264 genome, it became clear that this gene may be part of a small gene operon or gene cluster containing other genes potentially involved in PHA synthesis (Fig. 2a). These additional genes were identified as *phbA*, *phbB* and *phaR*, and the function of their products was computationally predicted using clusters of orthologous groups (COGs) of proteins. The *phbA* gene was found to code for acetyl CoA-acetyltransferase, the *phbB* gene was found to code for acetyacetyl CoA reductase while the *phaR* gene was found to code for an unknown protein; however, using BLASTx alignments, its function was predicted to be as a DNA-binding polyhydroxyalkanoate synthesis repressor.

It was clear from these results that the gene cluster identified could be potentially involved with the production of PHA in *B. thailandensis* E264. To investigate any specific metabolic pathways within which these genes played a major role, the gene products were analysed using the Kyoto Encyclopedia of Genes and Genomes (KEGG). KEGG analysis predicted that *phbA*, *phbB* and *phbC* have a role in the butyrate biosynthesis pathway in *B. thailandensis* E264. Specifically, the combination of these three gene products results in the formation of poly-β-hydroxybutyrate from acetyl CoA (Fig. 2b). The *phaR* gene was not found to be part of any metabolic KEGG pathways; this is understandable, however, if its main function is to act as a DNA binding-transcriptional repressor. In addition, *phbA*, coding for acetyl CoA-acetyltransferase, was also predicted to be involved in many other important metabolic pathways including different carbon metabolism pathways.

### Selection of *B. thailandensis* transposon mutants

Gallagher et al. (2013) reported the creation of a sequence-defined transposon mutant library within *B. thailandensis* E264 providing a wide range of single insertion transposon mutants for research purposes (Gallagher et al. 2013). Using this library, multiple transposon insertion mutants for *phbA*, *phbB* and *phbC* were identified and acquired (Supplementary Table S1). Transposon insertion into the gene of interest within each mutant was confirmed via PCR using primers external to the respective gene (data not shown). Using Sudan Black staining, the *B. thailandensis* transposon mutants were screened for PHA production. The clear observation of a reduction in bluish-black staining in the transposon mutant strain colonies when compared to the *B. thailandensis* E264 WT colonies indicated reduced PHA production in the transposon mutants (Supplementary Fig. S1).

### Fermentation analysis of *B. thailandensis* transposon mutants and initial screening of RL synthesis

The ten transposon mutant strains, WT *B. thailandensis* E264 and the RL-deficient *B. thailandensis rhlA* double mutant (annotated here as Δ*rhlA1*/*A2*) were cultured in shake flasks. Samples were taken every 24 h throughout the fermentation period to measure bacterial growth and every 48 h to measure glycerol depletion. End-point samples were taken after 264 h fermentation to quantify RL and PHA production.

*B. thailandensis* strains with transposon insertions in the same gene produced almost identical growth curve patterns and values (Supplementary Fig. S2). There was, however, a wide variation in the growth of the mutant strains with all transposon mutants producing OD_600_ values less than the WT strain throughout the fermentation period. The *phbC* mutants produced the least amount of biomass after 264 h fermentation ~1.82 g, with the *phbB* and *phbA* mutants producing ~3.06 and ~5.49 g, respectively, in comparison to the WT which produced 6.66 g. The Δ*rhlA1*/*A2* strain produced 7.85 g.

GC-MS was used to quantify glycerol concentrations in the medium throughout the fermentation period. There was no significant difference in glycerol depletion pattern between any of the *B. thailandensis* transposon mutant strains and the WT strain. Following 246 h incubation, all transposon mutant strains and the WT had a final glycerol concentration within the range of 1.28–1.94% (*w*/*v*) (Supplementary Fig. S3). The Δ*rhlA1*/*A2* strain showed a slower decline in glycerol concentration and had the highest residual concentration value of 2.04% (*w*/*v*) after 264 h incubation (Supplementary Fig. S3).

Gravimetric analysis of crude extracts obtained from each culture showed a variation in the range of RL production (g l^−1^) among the *B. thailandensis* transposon mutant strains for each of the three genes (Supplementary Fig. S4). Importantly, when compared with the WT, there was a significant increase in crude RL yields in all three *phbA* and *phbB* transposon mutant strains (Supplementary Fig. S4). This pattern was less conclusive in the *phbC* mutant strains, with two strains showing a non-significant trend towards increased RL production and two strains showing a significant trend towards reduced RL production when compared with the WT (Supplementary Fig. S4). The transposon mutant that produced the highest crude RL yield was Δ*phbB*1 with a yield of 3.99 g l^−1^ compared to 1.49 g l^−1^ in the WT. Based on these data, it was decided that the mutants with the highest crude RL yield for each gene would be further investigated; therefore, the *phbA*1, *phbB*1 and *phbC*1 mutants were used for all further analyses in this study.

### Quantitative analysis of RL and PHA production by *B. thailandensis* transposon mutants

**Fig. 3:**
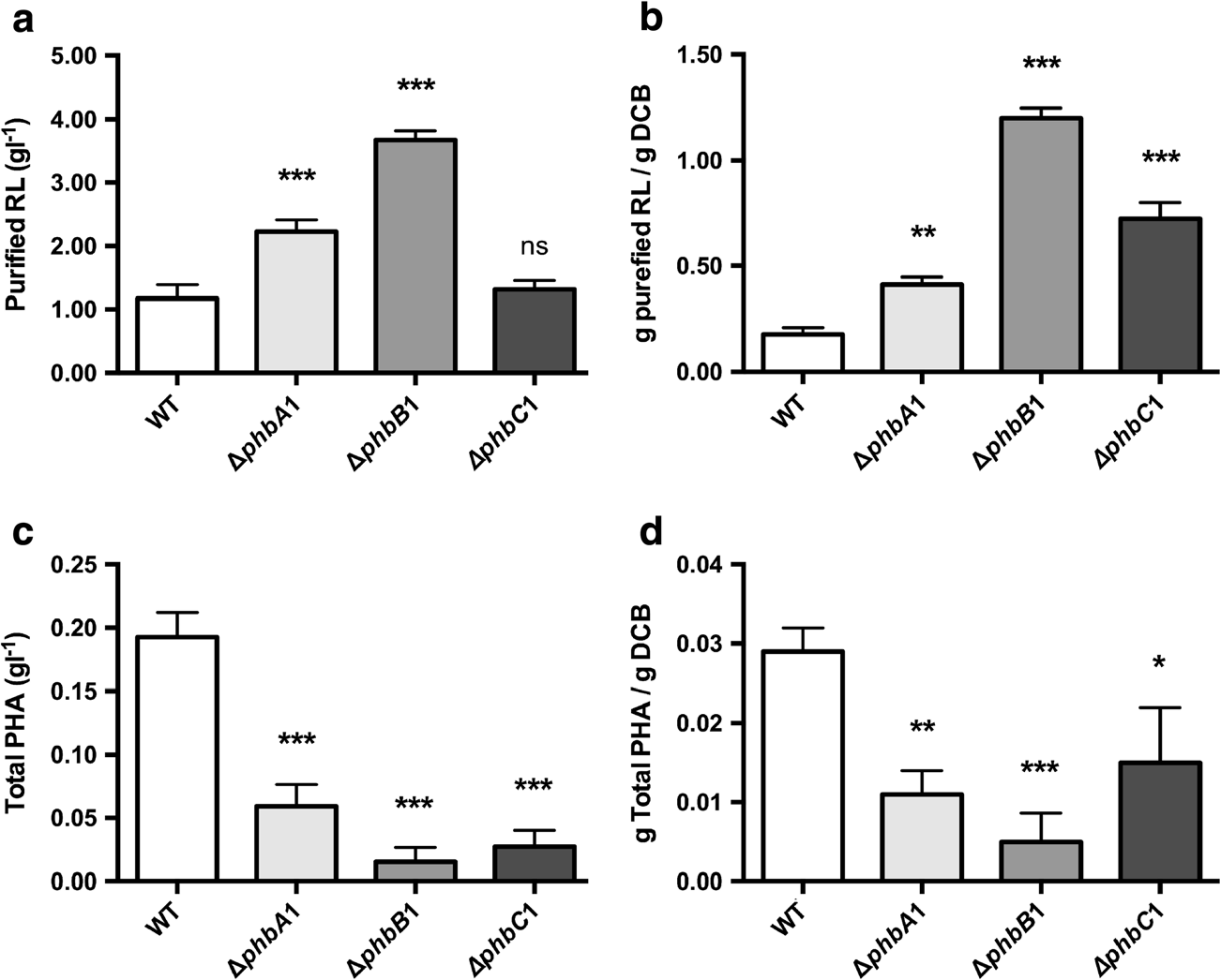
**a** Comparison of SPE-purified RL produced by *B. thailandensis* transposon mutants with the E264 WT. **b** Mean weights of purified RL normalised to dry cellular biomass. **c** GC-MS quantification of total PHA in *B. thailandensis* transposon mutant strains compared with the E264 WT. **d** Mean weights of total PHA normalised to dry cellular biomass. In all panels, the error bar represents standard deviation from the mean (*n* = 3 independent cultures). Data analysed using a one-way ANOVA with post hoc*.* Dunnett’s multiple comparisons tests (***p < 0.0001, **p < 0.01, *p < 0.05, ns = not significant)

To obtain a more accurate determination of RL production by the selected transposon mutant strains, solid-phase extractions (SPE) were carried out on the crude RL extracts to remove any contaminants or other cellular products that had been initially co-extracted with the RLs. The purified extract were then re-assessed gravimetrically. Any remaining non-RL contaminate was accounted for by subtracting the gravimetric measurement obtained from the non-RL-producing Δ*rhlA1*/*A2* strain from those obtained from the transposon mutants and WT. Both the *phbA*1 and the *phbB*1 mutants had a yield of purified RL that was significantly higher than that of the WT strain E264; however, RL production in the *phbC* mutant did not reach a level that was significantly increased in comparison to the WT (Fig. 3a). The *phbB*1 mutant showed the highest level of RL production with a yield of 3.67 g l^−1^ (± 0.15 g l^−1^) purified RL, while the WT strain had a yield of 1.17 g l^−1^ (± 0.22 g l^−1^) representing a 3.14-fold increase in RL production. Results from the other mutant strains showed that *phbA*1 produced 2.22 g l^−1^ (± 0.19 g l^−1^) purified RL and the *phbC*1 produced 1.32 g l^−1^ (± 0.14 g l^−1^). Interestingly, when purified RL yield for each of these three mutants was normalised to dry cellular biomass, it was found that all three mutant strains had a significantly higher RL production yield than the WT (Fig. 3b). The mean rate of RL production normalised to dry cell biomass across the total incubation time varied between 4.92 mg RL g^−1^ DCB per minute (Δ*phbB*1 mutant) and 0.76 mg RL g^−1^ DCB per minute (WT). GC-MS analysis of the WT strain and the transposon mutant strains *phbA*1, *phbB*1, *phbC*1 alongside a hexadecanoic acid standard allowed for direct quantification of the amount of PHA present in each sample. All of the transposon mutants produced significantly less PHA than the WT (Fig. 3c). This trend was further observed when PHA production was normalised to dry cellular biomass (Fig. 3d). Interestingly, PHA production although significantly reduced was not completely eliminated in the three transposon mutant strains.

### Qualitative analysis of RL production in *B. thailandensis* transposon mutants

**Fig. 4:**
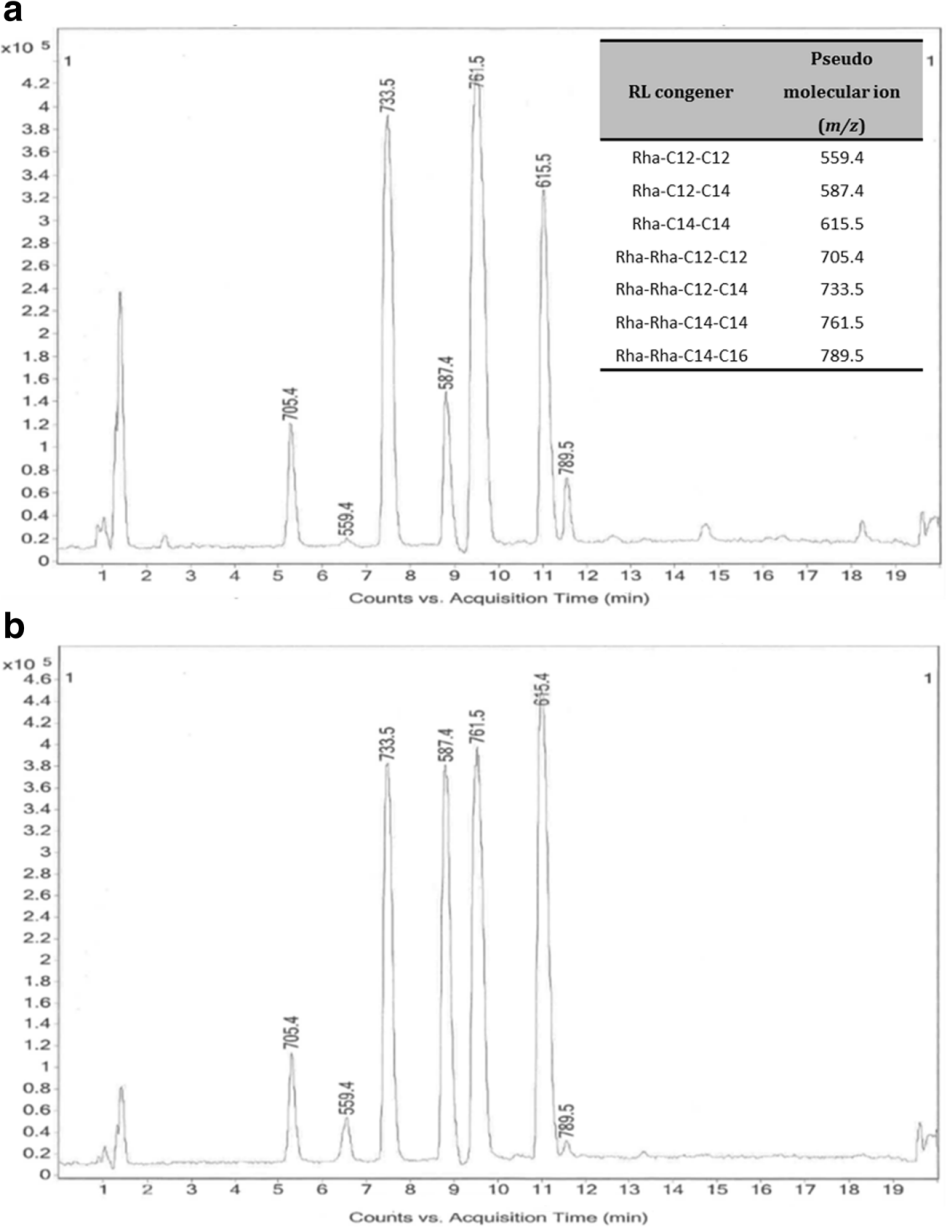
**a** HPLC-MS analysis of RLs produced by WT *B. thailandensis* E264. **b** HPLC-MS analysis of RLs produced the *B. thailandensis phbB*1 mutant strain

As the *phbB*1 mutant was shown to have the greatest increase in RL production compared to the WT strain, LC-MS analysis was carried out to examine if there were any differences in specific RL congeners produced (Fig. 4). Results showed that there was a significant shift in the ratio of mono-RL:di-RL produced by the *phbB*1 mutant compared to the WT strain (Table 1).

**Table 1:** Comparison of specific RL congener production between WT *B. thailandensis* and the PHA-deficient *B. thailandensis phbB*1 transposon mutant after 264-h fermentation in NB + 4% glycerol

Rhamnolipid congener	Pseudomolecular ion (*m*/*z*)	Retention time (min)	Relative abundance (%)	
			E264	*phbB*1
Rha-C12-C12	559.4	6.5	0.27	1.96
Rha-C12-C14/C14-C12	587.4	8.8	7.7	19.68
Rha-C14-C14	615.5	11	19.11	27.94
Rha-Rha-C12-C12	705.4	5.3	4.88	3.84
Rha-Rha-C12-C14/C14-C12	733.5	7.5	25.69	19.93
Rha-Rha-C14-C14	761.5	9.5	39.88	26.15
Rha-Rha-C14-C16/C16-C14	789.5	11.5	2.46	0.51

## Discussion

The main aims of this study were to establish the presence of a PHA synthesis system in *B. thailandensis* and to determine if the disruption of this system would lead to an increase in RL production by driving more carbon towards RL synthesis. Both PHAs and RLs are secondary metabolite compounds that are produced using similar biosynthetic pathways. In addition, they share some of the same precursor molecules meaning that, from a systems biology perspective, there is potential for process manipulation to increase RL production. Previous research carried out in *P. aeruginosa* was unable to demonstrate that nullifying PHA synthesis increased RL production (Choi et al. 2011). The lack of increased RL in *P. aeruginosa* PHA synthesis mutants was attributed to RL synthesis being stringently regulated in *P. aeruginosa* by a complex cell density-dependent QS system (Perfumo et al. 2013).

This study has demonstrated for the first time that *B. thailandensis* has a functional PHA synthesis system and is capable of producing PHA polymers and the monomeric constituents of these polymers. These data have also predicted one possible pathway and precursor molecules used by *B. thailandensis* for PHA synthesis and have shown that the genes responsible for this pathway reside in a single operon. Similar to RL synthesis, acetyl CoA is a major precursor for PHA synthesis, which is initially converted to acetoacetyl CoA by acetyl CoA-acetyltransferase encoded by *phbA*. Acetyl CoA-acetyltransferase is then reduced to R-3-hydroxybutanol-CoA by the product of the *phbB* which encodes for acetoacetyl CoA-reductase. The product of the *phbC*, poly(R)-hydroxyalkanoic acid synthase, class I, then converts R-3-hydroxybutanol-CoA to poly-β-hydroxybutyrate. At present, this is the only PHA biosynthesis system that has been identified in *B. thailandensis* and shown to be functional in a laboratory study.

To fully analyse the functionality of this PHA synthesis system, a number of transposon mutants were selected for each gene in the operon. Observations made during this study showed that the increase in acetyl CoA availability through the mutation of *phbA* resulted in a significant decrease in PHA production coupled with a significant but not substantial increase in RL synthesis and reduction in cell replication/growth rate. This indicates that although there was a significant reduction in PHA, the resulting free acetyl CoA was not efficiently driven towards RL production. The fundamental reason for this is that acetyl CoA is used for a wide range of cellular processes within the cell. Oh et al. (2014) demonstrated that in *Burkholderia*, acetyl CoA is used with oxaloacetate for the synthesis of citrate in the tricarboxylic acid (TCA) cycle, which is subsequently used as a precursor for oxalic acid production (Oh et al. 2014). Oxalic acid plays a very important role in the survival of *Burkholderia* upon entry to the stationary phase. Oxalic acid is produced by *Burkholderia* species through cell density-dependent QS systems which predict the onset of the stationary phase and subsequently counteract increasing pH levels caused by environmental ammonia accumulation, preventing significant population crashes (Goo et al. 2012). As this system plays such a significant role in *B. thailandensis*, it is possible that the majority of free acetyl CoA created by the mutation of *phbA* is utilised in oxalic acid synthesis and therefore there was no significant increase in RL production.

Knockout of the *phbB* gene proved to be the most optimal mutation for increasing RL production in *B. thailandensis* resulting in a 3.14-fold increase in RL yield for the *phbB*1 mutant (3.67 g l^−1^ purified RL compared to the WT strain 1.17 g l^−1^ purified RL). In addition, the Δ*phbB*1 strain showed a significant decrease in PHA production and a decrease in biomass. We postulate that mutation of the acetoacetyl CoA reductase-encoding *phbB* gene in *B. thailandensis* leads to an increase of acetoacetyl CoA within the cell resulting in successfully driving more carbon towards RL production compared with the *phbA* mutant. One explanation for this is that it could have been caused by high amounts of acetoacetyl CoA reaching a threshold and initiating downregulation of PHA production through the *phaR* gene. There is, however, limited evidence to support this in other bacteria as expression of the *phaR* gene seems to be initiated directly by poly (R)-3-hydroxybutyrate (Maehara et al. 2002). Another possible explanation is that unused acetoacetyl CoA was recycled through the fatty acid synthesis pathway and its subsequent components redirected towards RL production. The production of PHA was not completely nullified in any of the transposon mutants indicating the presence of additional PHA synthesis systems in *B. thailandensis* which may be functional and contribute to overall PHA accumulation potential genes including *phaC* homologues found on chromosome 2 (WP_009895308.1) and chromosome 1 (WP_009890861.1). Additionally, studies of PHA synthesis in *P. aeruginosa* have demonstrated that RhlA can aid in PHA synthesis (Soberón-Chávez et al. 2005; Cabrera-Valladares et al. 2006). *B. thailandensis* also possesses two identical RhlA homologues; therefore, a similar process may be occurring here.

The *phbC* mutant was hypothesised to have the greatest effect on increasing RL production. This was due to the product of the *phbC* gene, poly(R)-hydroxyalkanoic acid synthase, class I, being highly specific to PHA production with no evidence that it is involved in any other metabolic pathways in *B. thailandensis*. The mutation of this gene resulted in a significant reduction in cell growth and biomass accumulation coupled with a significant decrease in PHA; however, in contrast to the *phbA* and *phbB* mutants, there was no significant increase in RL production yield. The RL production yield of the best performing *phbC* mutant, *phbC*1, was on a similar level to that observed in the WT strain rendering the hypothesis invalid. There was, however, a significant increase in specific productivity of RL in the *phbC*1 mutant as the cell produced significantly more RL per gram of DCB than the wild type (0.72 g RL g^−1^ DCB compared to 0.18 g RL g^−1^ DCB). Therefore, although the overall level of RL produced was similar to that of the WT strain, the Δ*phbC*1 strain cells were producing RL at a level 4.00-fold higher than the WT strain.

These results contrast heavily with previous research carried out in *P. aeruginosa* where mutation of PHA synthesis did not drive increased RL production. This is a potential indication that RL production in B*. thailandensis* unlike in *P. aeruginosa* may not be as stringently regulated by cell density-dependent QS systems (Choi et al. 2011, Perfumo et al. 2013). These findings could have implications in further process optimisation of *B. thailandensis* for maximal RL production. This study has also shown that removing PHA production in *B. thailandensis* induced a large shift in the ratio of mono-RL:di-RL produced, resulting in an almost equal ratio of mono-RL:di-RL in the *phbB*1 mutant strain compared to the predominantly di-RL-producing WT strain. This increase in mono-RL may have been caused by the increase in free C-based precursors allowing for more HAA production. However, as the cells are still producing rhamnose at the same rate as the WT, there may not be enough for the efficient conversion of mono-RL to di-RL that is seen in the WT strain where less overall RL is produced.

These findings show that the flux of closely related metabolic pathways can be manipulated to re-route specific resources towards a desired product. Another interesting observation from this study was that the glycerol consumption of the PHA transposon mutant strains was not significantly different from that of the WT strain or the RL-negative Δ*rhlAD* strain. Confirming that although the growth kinetics and metabolite production differ between strains, similar amounts of glycerol are consumed during the fermentation process indicating that different ratios of metabolic products are formed. What is clear is that metabolic flux may be a key area in successfully creating a *B. thailandensis* mutant strain that can produce high levels of RL. Further work in this area could be to attempt to knockout the oxalic acid synthesis pathway in addition to the PHA synthesis pathway to free up more fatty acid precursor for RL synthesis. While this would, however, require the culture pH to be closely maintained externally, it could potentially lead to a further increase in RL production.

## Electronic supplementary material

ESM 1 (PDF 651 kb)
