# Supplementary material for: Enhanced rhamnolipid production in Burkholderia thailandensis transposon knockout strains deficient in polyhydroxyalkanoate (PHA) synthesis
Source: Appl Microbiol Biotechnol. 2017 Oct 17;101(23):8443–54. doi: 10.1007/s00253-017-8540-x (PMC5694511; doi:10.1007/s00253-017-8540-x)
Supplement: Supplementary file 1 — (PDF 651 kb) [file 253_2017_8540_MOESM1_ESM.pdf]

APPLIED MICROBIOLOGY AND BIOTECHNOLOGY

Supplementary material for:

Enhanced rhamnolipid production in *Burkholderia thailandensis* transposon knockout strains deficient in polyhydroxyalkanoate (PHA) synthesis

Scott J Funston[1] - Konstantina Tsaousi[1] –Thomas J Smyth[2] - Matthew S. Twigg[1] - Roger Marchant[1] – Ibrahim M Banat[1*]

[1]School of Biomedical Sciences, Ulster University, Coleraine, BT521SA, Northern Ireland, UK

[2]Department of Life Sciences, Institute of Technology Sligo, County Sligo, Ireland

Corresponding author: Ibrahim Banat

im.banat@ulster.ac.uk tel:+44287012 3062 fax:+44287012 4965/3159

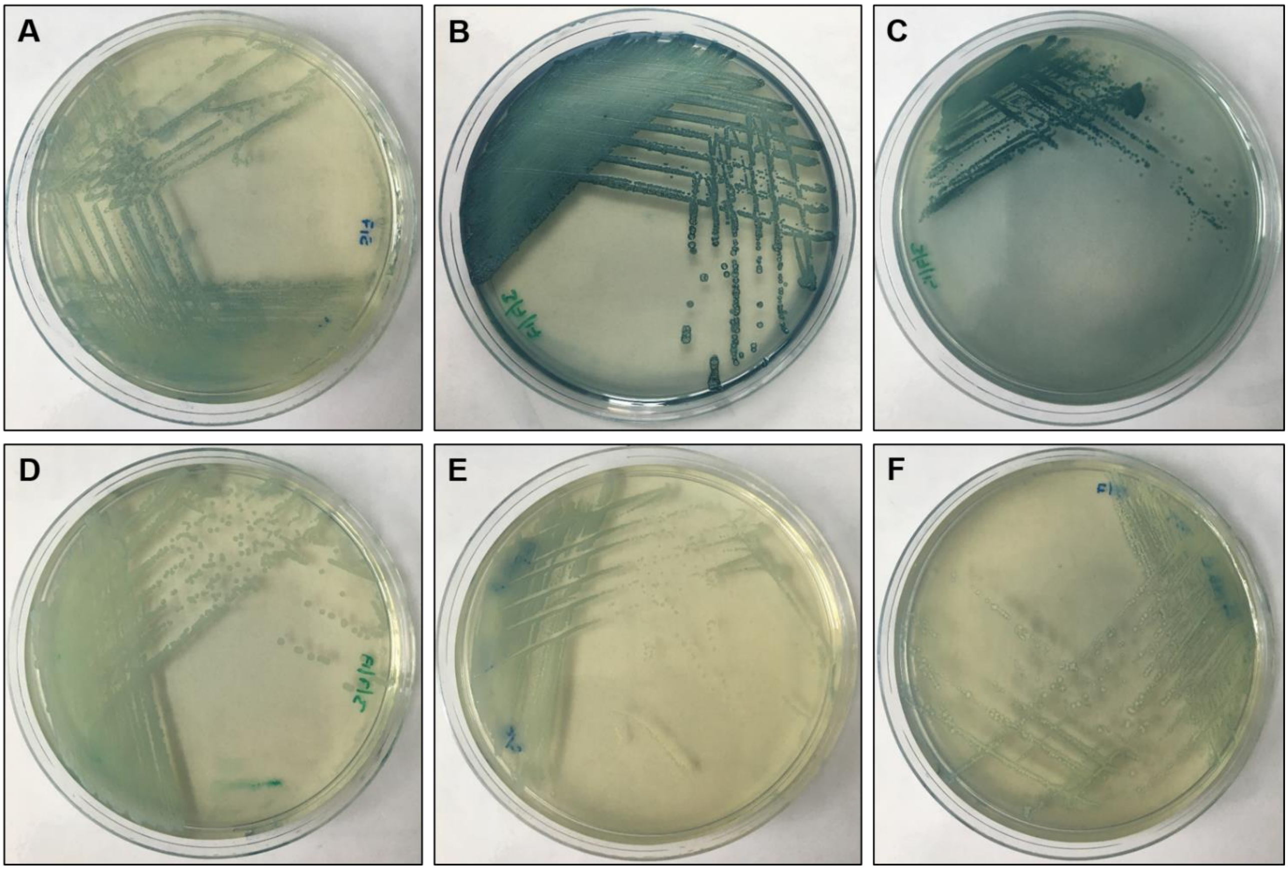

Figure S1

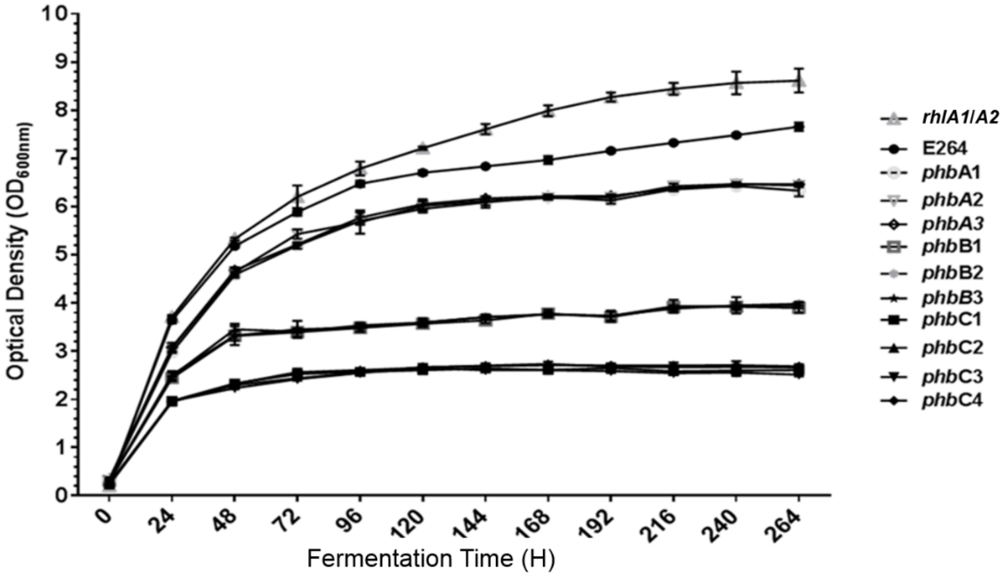

Figure S2

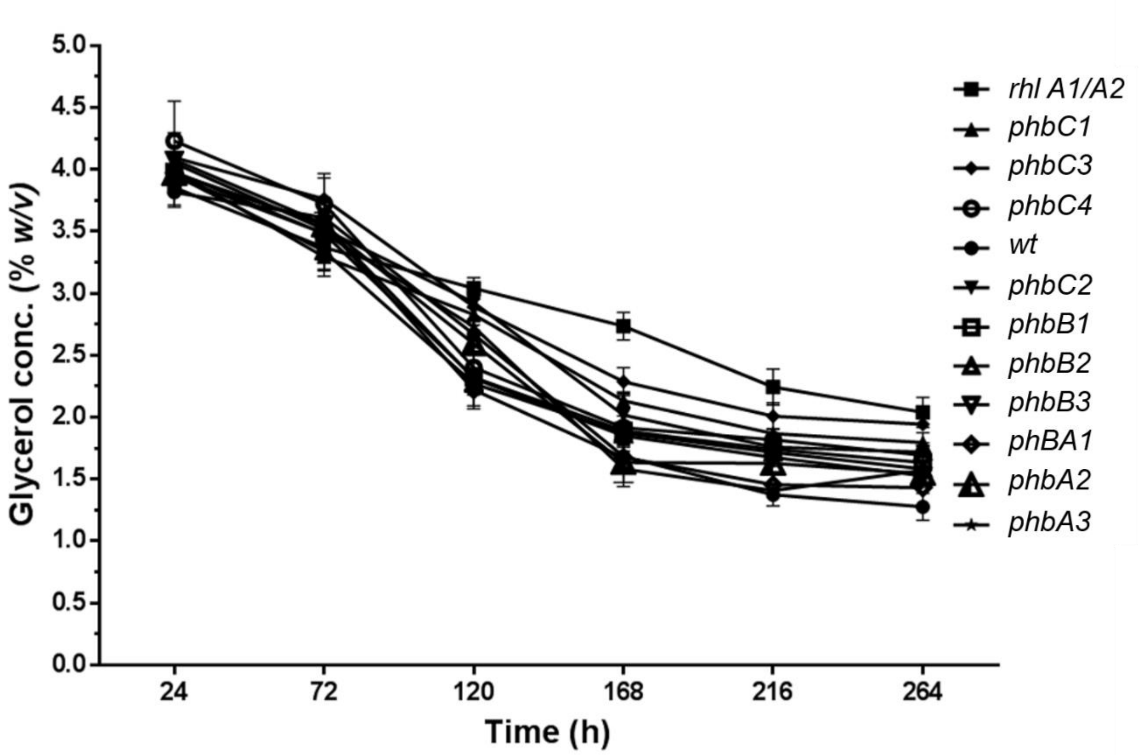

Figure S3

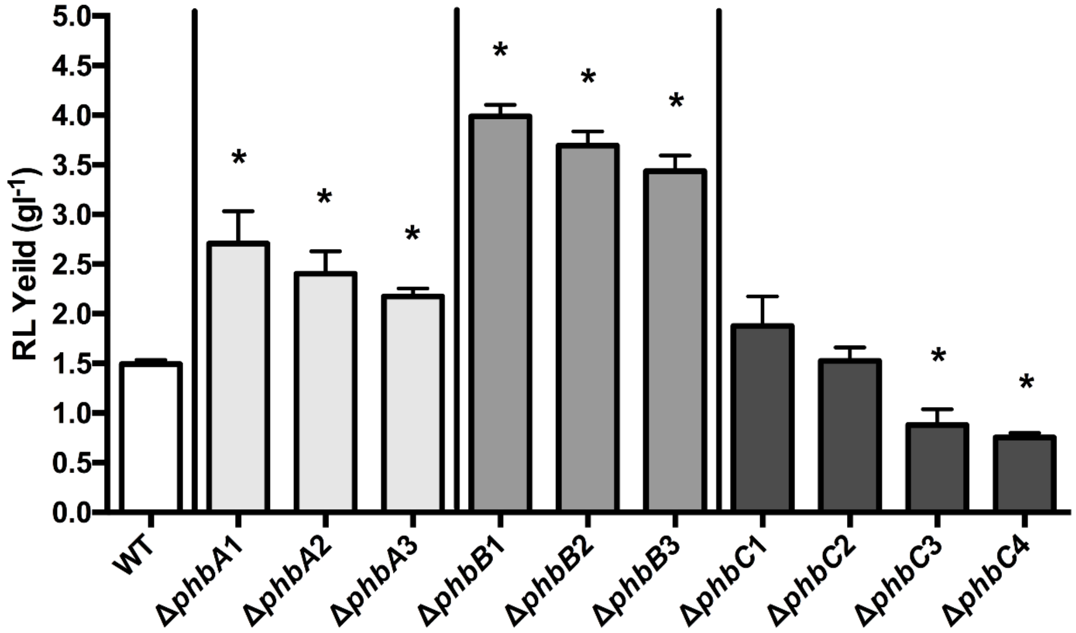

Figure S4

Table S1. List and description of *B. thailandensis* transposon mutants in PHA synthesis genes used in this study.

Species	Strain Name	Strain Description	Transposon Mutant Library Designation (Gallagher *et al.* 2013)
B. thailandensis	E264 *phbC*1	T23 transposon mutant in BTH_I2255 (*phbC*) encoding a poly(R)-hydroxyalkanoic acid synthase, class I	tnbt1_er100504p04q148
B. thailandensis	E264 *phbC*2	T23 transposon mutant in BTH_I2255 (*phbC*) encoding a poly(R)-hydroxyalkanoic acid synthase, class I	tnbt1_er100421p06q104
B. thailandensis	E264 *phbC*3	T23 transposon mutant in BTH_I2255 (*phbC*) encoding a poly(R)-hydroxyalkanoic acid synthase, class I	tnbt1_er100421p06q152
B. thailandensis	E264 *phbC*4	T23 transposon mutant in BTH_I2255 (*phbC*) encoding a poly(R)-hydroxyalkanoic acid synthase, class I	tnbt1_er100408p04q145
B. thailandensis	E264 *phbA*1	T23 transposon mutant in BTH_I2256 (*phbA*) encoding a acetyl-CoA acetyltransferase	tnbt1_er100412p05q191
B. thailandensis	E264 *phbA*2	T8 transposon mutant in BTH_I2256 (*phbA*) encoding a acetyl-CoA acetyltransferase	tnbt1_2r100310p04q118
B. thailandensis	E264 *phbA*3	T23 transposon mutant in BTH_I2256 (*phbA*) encoding a acetyl-CoA acetyltransferase	tnbt1_er100329p06q190
B. thailandensis	E264 *phbB*1	T23 transposon mutant in BTH_I2256 (*phbB*) encoding a acetyacetyl-CoA reductase	tnbt1_er100329p01q164
B. thailandensis	E264 *phbB*2	T23 transposon mutant in BTH_I2256 (*phbB*) encoding a acetyacetyl-CoA reductase	tnbt1_er100406p01q116
B. thailandensis	E264 *phbB*3	T23 transposon mutant in BTH_I2256 (*phbB*) encoding a acetyacetyl-CoA reductase	tnbt1_er100419p05q185

Supplementary Figure Legends

Figure S1. PHA synthesised within the cell was stained using Sudan Black. A. *E. coli* JM109 (PHA -). B. *P. aeruginosa* PAO1 (PHA +). C. *B. thailandensis* E264 WT. D. *B. thailandensis* E264 Δ*phbA*1. E. *B. thailandensis* E264 Δ*phbB*1. F. *B. thailandensis* E264 Δ*phbC*1

Figure S2. Growth analysis of *B. thailandensis phb* transposon mutants, *B. thailandensis* E264 WT and *B. thailandensis rhlA1/A2* throughout a 264 h fermentation period. Mean values with error bars representing standard deviation from the mean (n=3).

Figure S3. Glycerol concentrations of *B. thailandensis phb* transposon mutants, *B. thailandensis* E264 WT and *B. thailandensis rhlA1/A2* throughout a 264 h fermentation period. Values were obtained using GC-MS. Mean values with error bars representing standard deviation from the mean (n=3).

Figure S4. RL production in *B. thailandensis* transposon mutants after 264 h fermentation in shake flasks. Mean weights of crude RL extracts from three replicate cultures with error bars representing SD in weight from the mean. Data analysed using a one-way ANOVA with *post hoc.* Tukey's multiple comparisons tests, (* $p < 0.05$).
